# An appraisal of whole-room indirect calorimeters and a metabolic cart for measuring resting and active metabolic rates

**DOI:** 10.1038/s41598-020-71001-1

**Published:** 2020-08-31

**Authors:** Shanshan Chen, Cory Scott, Janina V. Pearce, Jared S. Farrar, Ronald K. Evans, Francesco S. Celi

**Affiliations:** 1 Department of Biostatistics, School of Medicine, Virginia Commonwealth University, Richmond, USA; 2 Division of Endocrinology Diabetes and Metabolism, Department of Internal Medicine, School of Medicine, Virginia Commonwealth University, 1101 East Marshall Street, Sanger Hall, Room 7-007, PO Box 980111, Richmond, VA 23298-0111 USA; 3 Department of Kinesiology and Health Sciences, Virginia Commonwealth University, Richmond, USA

**Keywords:** Metabolism, Obesity

## Abstract

Whole-room indirect calorimeters (WRICs) have traditionally been used for real-time resting metabolic rate (RMR) measurements, while metabolic rate (MR) during short-interval exercises has commonly been measured by metabolic carts (MCs). This study aims to investigate the feasibility of incorporating short-interval exercises into WRIC study protocols by comparing the performance of WRICs and an MC. We assessed the 40-min RMR of 15 subjects with 2-day repeats and the 10–15 min activity MR (AMR) of 14 subjects at three intensities, using a large WRIC, a small WRIC, and an MC. We evaluated the biases between the instruments and quantified sources of variation using variance component analysis. All three instruments showed good agreement for both RMR (maximum bias = 0.07 kcal/min) and AMR assessment (maximum bias = 0.53 kcal/min). Moreover, the majority of the variability was between-subject and between-intensity variation, whereas the types of instrument contributed only a small amount to total variation in RMR (2%) and AMR (0.2%) data. In Conclusion, the good reproducibility among the instruments indicates that they may be used interchangeably in well-designed studies. Overall, WRICs can serve as an accurate and versatile means of assessing MR, capable of integrating RMR and short-interval AMR assessments into a single protocol.

## Introduction

Whole-room indirect calorimeters (WRICs) have been used to assess various metabolic states in both healthy subjects and subjects affected by pathologies^1,2^. A WRIC, an isolated room with a known volume and a controlled air inflow rate, provides minute-by-minute measurements of the subject’s O_2_ consumption and CO_2_ production from breaths, via the continuous measurement of O_2_ and CO_2_ concentrations in inflow air and outflow air. These data enable precise calculations of metabolic rate (MR, unit: kcal/min) for prolonged periods allowing for activities of daily living^3^, and energy expenditure (EE, unit: kcal), which is the cumulative MR over time. Hence, MR and EE in the general sense are used interchangeably in the medical literature and in this paper.

Human EE is comprised of various components and is influenced by many factors. The largest component of human EE is resting EE, which is the energy required to carry out fundamental physiological functions, contributing 60–80% of the total daily EE^4^. RMR is influenced by various physiological characteristics, including gender^5^, ethnicity^6^, age^7, 8^, body composition^9,10^, various metabolic syndromes^11–16^, and gene variations^17^. Additionally, resting MR (RMR) responds to environmental stimuli, such as cold temperatures^18,19^, food intake and dietary composition^20–24^. Moreover, RMR drops significantly during sleep^25^ and varies by circadian phase^26^. A smaller, yet important component of human MR is activity-induced MR (AMR)^27^. Activities can be subdivided into two categories: non-exercise activity thermogenesis (NEAT) and volitional exercise^28,29^. NEAT includes occupational and leisure activities and any spontaneous activities, such as fidgeting and maintenance of posture. Because of these factors, RMR needs to be assessed under controlled experimental conditions. By providing environmental control and real-time measurements over extended periods, WRICs are the perfect tools to isolate the various components of MR.

Due to the cost of building, maintaining, and operating WRICs, most research aimed at assessing MR has used less costly metabolic carts (MCs). Instead of placing the subjects in a room, MCs require the volunteer to lie under a ventilated hood for RMR measurements, or to wear a mouthpiece and nose clip, or a face mask while tethered to the system^30–32^. This may cause claustrophobic sensations in some subjects, potentially impacting RMR measurements^30^ and limiting the recording duration. For researchers who are interested in assessing MR over a range of activities, MCs can be difficult to maneuver because of the tethering and may alter normal breathing patterns^30–32^. Lastly, to assess both RMR and AMR during a single study visit, MCs would require additional time for equipment changes and system re-calibration.

Although WRICs provide researchers with more flexibility to study the effects of sedentary behavior^33^ and various activity types and intensities on total MR^34–38^, the “dilution effect” caused by the room size of the WRICs^3^ limits their temporal resolution, which has traditionally hindered their use in short-interval exercise studies. To remedy the long delay imposed by the room size of WRICs, we have recently devised a method to improve the temporal resolution of WRICs and validated our system against 22 sessions of 24-h gas infusion studies of dynamic metabolic profiles ^3^. Here, we further evaluate our system using data collected from 29 human subjects under both resting and exercising conditions, investigate the biases between instruments, and quantify the sources of variation in the MR mesurements.

## Methods

### Data collection

This study was conducted on 29 healthy, non-smoking subjects. Exclusion criteria included age < 18 years, medications affecting metabolism, pregnancy or lactation, reported claustrophobia, and resting blood pressure > 140/90 mmHg. RMR and AMR were measured using three instruments: a large WRIC (26 m^3^), a small WRIC (5.5 m^3^), and an MC. The two WRICs had been previously validated using gas-infusion methods^3^. The MC used in this study (Parvo Medics TrueOne 2400) was in an adjacent room approximately 20 m away from the WRICs. All instruments were calibrated by following the procedures below. Written informed consent was obtained prior to the first study visit and all study procedures related to human subjects were approved by the Institutional Review Board at Virginia Commonwealth University. All research was performed in accordance with the relevant guidelines and regulations.

### Instrument calibration

#### Calibration of WRICs

To ensure accurate measurements, the indirect calorimeters were calibrated prior to each testing session by following two procedures: (1) gas analyzer (manufactured by Siemens, model: Ultramat/Oxymat 6.) calibration using mixed gases, and (2) WRIC system calibration using a gas infusion method. For the first calibration procedure, reference points for calibration were obtained by mixing gases (N_2_, O_2_, and CO_2_) onsite to 10 known concentration levels, with O_2_ ranging from 20.0–21.0% and CO_2_ ranging from 0.0–1.0%. Gas mixing was automatically performed by a gas blender comprised of mass flow controllers (MFCs). Each MFC was pre-validated against a primary flow standard (ML-800; Mesa Laboratories, Butler, NJ). During the calibration stage, three MFCs were used to regulate the flow rates of N_2_, O_2_, and CO_2_. These three gases subsequently flowed into a manifold that mixed them at a known combination of flow rates, from which reference values for the O_2_ and CO_2_ concentrations were calculated. For the second calibration procedure, N_2_ and CO_2_ were infused into an empty chamber to simulate a human subject’s effect on the system and obtain reference values and the in silico performance of the WRICs. Flow rates of N_2_ and CO_2_ were pre-determined to generate reference values for volume of O_2_ (VO_2_) and volume of CO_2_ (VCO_2_), from which the reference values of MR were calculated via the Weir Equation^39^. The critical parameters for calculating VO_2_ and VCO_2_ (e.g. room volume, offsets between the input air analyzer and the room air analyzer) were estimated given the measurements and the reference MR. These critical parameters were then used in processing the raw data collected in the human studies. All gas samples during the studies were dried below 1,000 ppm using a gas sample dryer (manufactured by Perma Pure LLC, Lakewood, NJ). Other operational details of the WRICs have previously been published^3^.

#### Calibration of the Parvo metabolic cart

Prior to each RMR visit and AMR visit, the Parvo Medics TrueOne 2400 was allowed to warm up for a minimum of 30 min. Temperature, barometric pressure, and relative humidity were recorded to ensure accurate calibration of the instrument. Flow calibration was accomplished with a 3-L syringe with an error of ± 1%. Prior to each trial, O_2_ and CO_2_ analyzers were calibrated per manufacturer specifications with the following known gas mixtures: 1.004% CO_2_, 16.01% O_2_, balance N_2_ (RMR), and 4.000% CO_2_, 16.00% O_2,_ balance N_2_ (AMR).

### Study protocol

To minimize the confounding effects of natural variations in MR, we randomized the sequence of measurement instruments (a large WRIC, a small WRIC, or an MC) to be used for each subject. For RMR measurements, we repeated the experiment on separate days for each subject to allow the assessment of test–retest reliability. For AMR measurements, we devised individualized exercise intensities to ensure that subjects reached a steady-state MR.

#### Resting MR study

The RMR study consisted of two visits. To minimize biological variations in RMR, the two visits were scheduled for 2 days within 1 week, and female subjects were scheduled in the early follicular phase of their menstrual cycle (days 2–10). During each visit, the subject underwent RMR tests using all three instruments. The subjects were asked to arrive early in the morning (7:30 am—9:00 am) after an overnight fast (i.e. no food or caffeine intake) and no strenuous exercise for at least 24 h. A brief physical examination was also performed by a study physician to ensure that the inclusion and exclusion criteria were met. Following the physical examination, the subjects were asked to lie in a supine position for approximately 40 min and refrain from sleeping or moving. Subjects were also observed and kept awake during the trials. The room temperature was controlled at 24 ˚C.

#### Active MR study

The AMR study consisted of three visits. The three visits were scheduled for three days within one week, with at least 24 h between each visit. The precautionary steps taken to eliminate biological variation in AMR were similar to the RMR tests, except that subjects could choose to arrive either early in the morning after overnight fasting (7:30 am–9:00 am) or late afternoon (3:00 pm–4:00 pm) after at least a 4-h fast, and this was held constant for each subsequent visit. After a routine examination, the subjects completed a 60-min exercise session on a cycle ergometer (VIAsprint 150P or Monark 928E) using one of the three randomly-assigned instruments. The same ergometer was used for all three visits for each participant. The three exercise intensities for each visit were determined based on the subject’s weight and gender. The workloads of the three intensities were 0.75, 1.5, and 2.25 W/kg for males, and 0.5, 1.0, and 1.5 W/kg for females. Each subject exercised for 15 min at each of the first two intensity levels, and 10 min at the highest intensity level, with a 10-min period of rest between each level. This exercise protocol was designed to account for the gender difference in power output and provide sufficient recovery time between each short-interval exercise in order to minimize fatigue.

### Statistical analysis

We performed data analysis in Matlab 2019b (Mathworks Inc, Natick, Massachusetts) and R Studio (RStudio Inc., Boston, Massachusetts). The resting MR (RMR) was the average of a 40-min MR recording during supine rest, with the initial 10 min before the WRICs reached equilibrium discarded, resulting in 30 min of steady-state RMR. For the AMR measurements, we manually selected the steady-state MR, defined as the flat region between transition edges after plotting the entire session in Matlab. This resulted in approximately 12 min of data for the first two intensity levels and approximately 7 min of data for the highest intensity level, and we averaged the steady-state MR at each intensity.

In our analysis, we first assessed biases between instruments at each visit or at each exercise intensity level, and tested the group differences using two-sided, paired t-tests. To visualize these biases, we also generated Bland–Altman plots for all comparisons. Test–retest reliability was assessed using the Pearson correlation coefficient between the RMRs measured on two visits. To check the validity of our exercise protocol, we assessed the intensity range using metabolic equivalent of tasks (METs, unit: kcal/h/kg) for each subject^40^. Since the true AMR is unknown, we assessed the linear response of the three instruments to various workloads as a proxy for instrument accuracy. Lastly, to assess the variability in MR between instruments over successive visits or at each exercise intensity, we conducted variance component analyses^41^ to decompose the total variance in the data into the percentage contributions of various factors (i.e. instrument type, subject, visits, and intensity levels).

## Results

Of the 29 subjects, 15 participated in the RMR trial and 14 participated in the AMR trial (Table 1). A variability plot showing each subject’s measurements and Bland–Altman plots to illustrate the biases are in Supplementary Information Figures S1–S3.

**Table 1:** Subject anthropometric data for the resting energy expenditure study (RMR) and the exercise study (AMR).

	Gender	Age (years)	Height (cm)	Weight (kg)	BMI (kg/m^2^)
RMR (15 subjects)	9 males, 6 females	31.5 (12.4)	172.0 (10.6)	74.3 (16.2)	24.9 (3.8)
AMR (14 subjects)	9 males, 5 females	37.4 (15.9)	172.6 (7.4)	72.5 (9.8)	24.3 (2.4)

**Figure 1:**
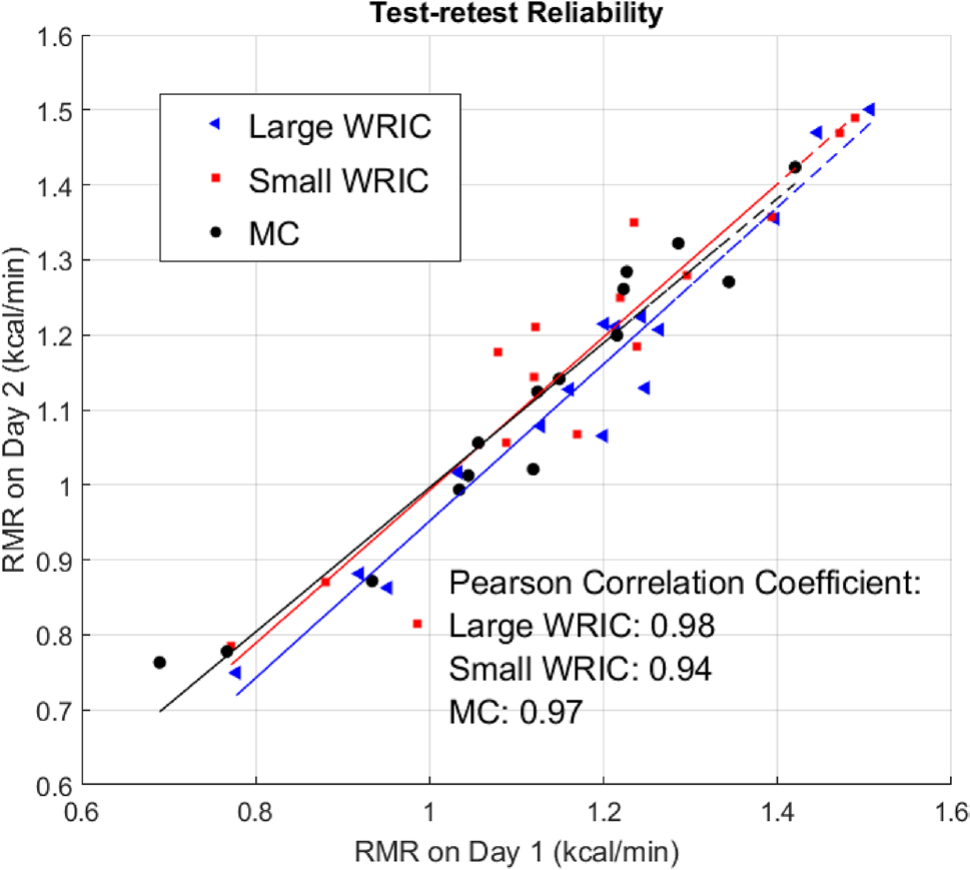
Test–retest reliability of the three instruments.

Compared with the WRICs, the MC tended to underestimate RMR, showing an average bias of -0.05 kcal/min compared with the large WRIC and an average bias of − 0.06 kcal/min compared with the small WRIC. Measurements from the large WRIC and small WRIC agreed well, but the values obtained from the large WRIC were slightly lower than those from the small WRIC, with a bias of − 0.01 kcal/min (Table 2). Moreover, all three instruments showed excellent test–retest reliability, assessed by Pearson correlation coefficient, as shown in Fig. 1.

**Table 2:** Biases in RMR measurements between the three instruments.

		Large WRIC vs MC	Small WRIC vs MC	Large WRIC vs small WRIC
Day 1	Bias ± SE (kcal/min)	0.07 ± 0.00 (p = 0.001)	0.06 ± 0.00 (p = 0.002)	0.01 ± 0.00 (p = 0.545)
	Relative error (%)	7.4%	7.2%	4.0%
	RMSE (kcal/min)	0.10	0.09	0.06
Day 2	Bias ± SE (kCal/min)	0.04 ± 0.00 (p = 0.019)	0.07 ± 0.01 (p = 0.005)	− 0.03 ± 0.00 (p = 0.130)
	Relative error (%)	4.8%	7.8%	3.9%
	RMSE (kcal/min)	0.07	0.10	0.07
Average	Bias ± SE (kcal/min)	0.05 ± 0.02	0.06 ± 0.00	− 0.01 ± 0.03
	Relative error (%)	5.8%	6.5%	2.3%

*p-values were obtained using two-sided, paired t-tests.

**Figure 2:**
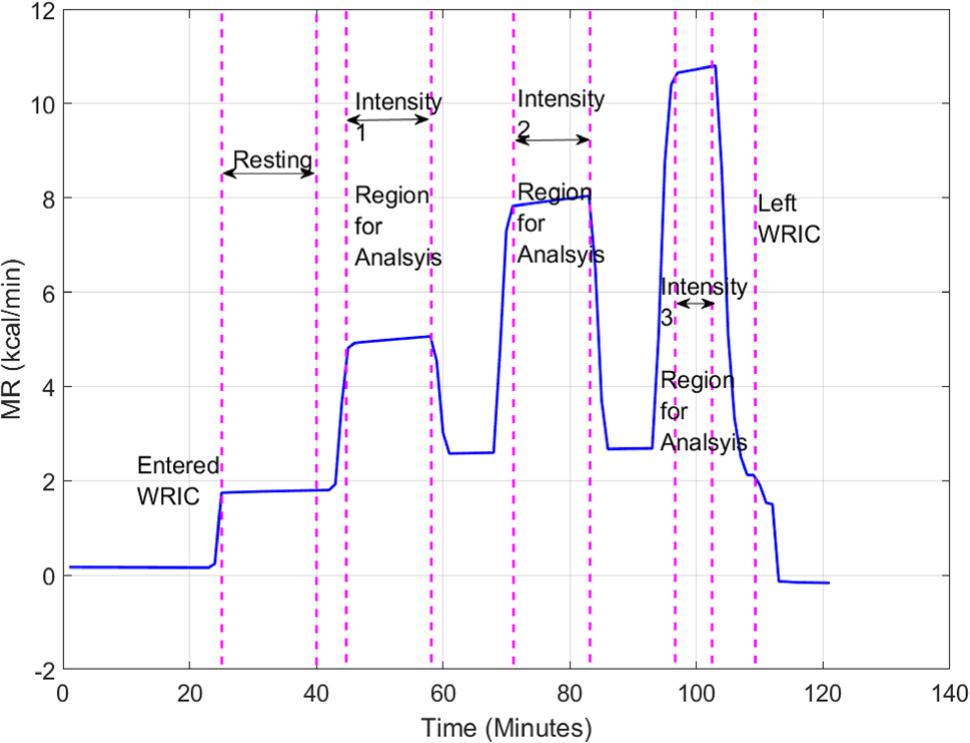
MR levels during one AMR session in the large WRIC. The blue line is the time series of MR for one AMR session.

Figure 2 illustrates that our AMR protocol and the previously-validated method for improving the temporal resolution of the WRICs^3^ were successful in capturing the fast-changing dynamics of exercise MR in the WRICs. The steady states at the three intensity levels were clearly delineated by the 10-min rest intervals using our previously published methods^3^. Comparisons of the agreement between instruments at each exercise level are listed in Table 3, and illustrated by the Bland–Altman plots in Supplementary Information Figure S5. The intensity range of the AMR protocol is shown in Supplementary Information Figure S6. Overall, the highest disagreements were between the large WRIC and the MC, with relative errors of about 10% across the three exercise levels. The small WRIC and the MC were in closer agreement, with relative errors of about 7.5%.

**Table 3:** Biases in exercise MR measurements between the three instruments.

		Large WRIC vs MC	Small WRIC vs MC	Large WRIC vs small WRIC
Intensity 1	Bias ± SE (kCal/min)	− 0.20 ± 0.06	0.10 ± 0.03	− 0.30 ± 0.06
	Relative error (%)	11.2%	7.6%	7.5%
	RMSE (kCal/min)	0.79	0.37	0.84
Intensity 2	Bias ± SE (kCal/min)	− 0.42 ± 0.06	− 0.10 ± 0.05	− 0.32 ± 0.03
	Relative error (%)	9.9%	7.5%	5.0%
	RMSE (kCal/min)	0.88	0.71	0.55
Intensity 3	Bias ± SE (kCal/min)	− 0.53 ± 0.08	− 0.37 ± 0.07	− 0.15 ± 0.04
	Relative error (%)	9.9%	7.4%	3.9%
	RMSE (kCal/min)	1.25	1.00	0.52

**Figure 3:**
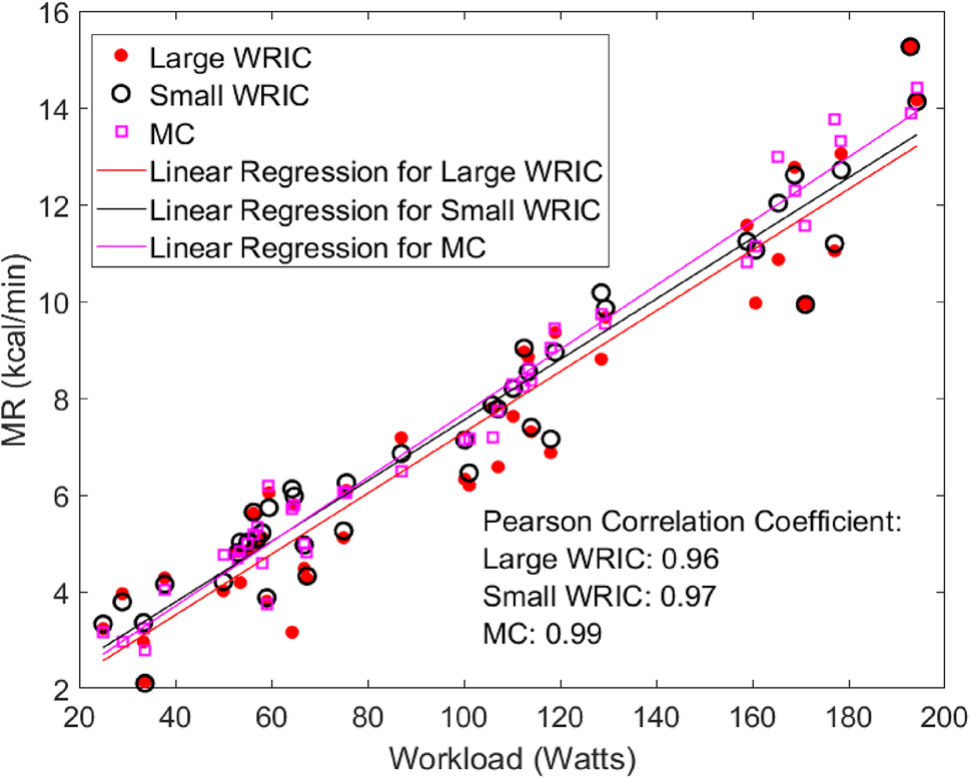
Regressions between workloads and MR measurements from the AMR study.

As we cannot measure AMR using any of the two instruments simultaneously, we modeled the relationship between exercise workloads and MR measurements from all three instruments, using the best linear response to workloads as a proxy for accuracy (Fig. 3). The MR measurements from the three instruments all show good linear correlations with the prescribed workloads, suggesting that the WRICs can be used for exercise tasks with various workloads. MRs measured by the MC show the best correlation with workloads, suggesting that an MC is a better choice than a WRIC for assessing AMR during a single bout of physical activity. However, the biases between them are tolerably small, and the versatility of WRICs can be of great benefit in studies of AMR across a range of activities over longer assessment periods.

Table 4 shows that the main source of variation in the RMR data was between subjects (92.7%), with only a small variation between instruments (2%). In contrast, when assessing AMR, the variation attributable to the type of instrument was only 0.2%, while the majority of the variation was attributable to the intensity levels (56.5%) and the between-subject variation (33.5%). As we prescribed individualized workloads for each intensity level, this large between-subject variation reduced to 4.5% once workload was considered as the main source of variation in lieu of intensity levels. In each case, the variation contributed by the types of instrument was equally small (0.2%).

**Table 4:** Results of the variance component analysis.

RMR study		AMR study			
Sources of variation	Percent (%)	Sources of variation	Percent (%)	Sources of variation	Percent (%)
Subject	92.7	Intensity levels	56.5	Workload	94.0
Instrument	2.0	Instrument	0.2	Instrument	0.2
Within-subject, between-visits	0.4	Subject	33.5	Subject	4.5
Residual	4.9	Residual	9.8	Residual	1.3

## Discussion

Our study evaluated the accuracy and consistency of WRICs for assessing RMR and AMR over a wide range of exercise intensities. Compared to an MC, our WRICs showed good consistency in both the RMR and AMR trials. This finding renders the WRIC a valid tool for recording MR during complex and dynamic protocols involving physical activities of various intensities, as well as RMR assessment, in one setting.

The RMR readings from both WRICs in the current study were higher than the values obtained via the MC. Similar findings were observed in a study by Rising et al.^42^, where an MC from a different manufacturer (Vmax Encore 2900, Carefusion Inc) also measured 10% lower RMR than a WRIC (bias = 0.14 kcal/min). The authors posited that the finding was due to the lack of adjustment for moisture in the gas samples in the MC system, which resulted in underestimation of the VO_2_ and VCO_2_. Unlike the WRIC, the ventilated hood method assumes constant environmental factors (e.g. air composition, presence of study personnel in the room, etc.) using a “reference air” canister as the ground truth which, if not tightly controlled, can critically affect the accuracy of the instrument^31^. In contrast, our WRIC systems actively dry the gas samples, isolate the influence of incoming air and take the gas concentration of the incoming air into account, leading to more accurate and precise measurements.

On average, our large WRIC systems measured about 5% lower than the MC across all exercise intensities, in contrast to the study by Rising et al.^43^, where they found that the energy expenditure assessed with their WRIC system was 30% greater than with their MC system (Vmax Encore 2900, Carefusion Inc). As we conducted our exercise test at much shorter intervals (10–15 min at each intensity level as opposed to 30 min at one intensity level), we suspect the response time of the WRICs might be responsible for the slight underestimation of AMR. This underestimation is reduced in the small WRIC due to its smaller volume and shorter response time.

Our variance component analyses show that reproducibility across the three instruments was excellent, contributing only 2% of the total variation in the RMR data and 0.2% in the AMR data. This suggests that when assessing cross-sectional RMR, the variation introduced by interchanging the three instruments could be negligible in studies investigating factors (e.g. gender, body composition, metabolic syndromes) that play a larger role. Moreover, in studies where AMR across activity intensities from low to vigorous activities is of interest, the three instruments can be used interchangeably if necessary.

Overall, our study demonstrates that modern WRICs can be used for studies involving both RMR and exercise measurement, by adequately capturing both in a single setting. Using our previously-validated methods for recovering dynamic WRIC signals^3^, our WRICs can capture short-interval exercises (10–15 min), which greatly complement their traditional role as a real-time RMR measurement tool. WRICs could facilitate the recording of AMR, since there is no need to fit mouthpieces and noseclips, substantially reducing the discomfort experienced by study participants. As assessing and modeling MR in free-living situations becomes increasingly important for clinical nutritional research and obesity research, the flexibility provided by accurate WRICs will allow for more complex study designs to better characterize near “free-living” conditions. Collectively, our study demonstrates that modern WRICs can be used as excellent research tools for studying the dynamics of human energy expenditure during both rest and exercise.

## Supplementary information

Supplementary Information 1.

Supplementary Information 2.

Supplementary Information 3.
