# Supplementary material for: An appraisal of whole-room indirect calorimeters and a metabolic cart for measuring resting and active metabolic rates
Source: Sci Rep. 2020 Aug 31;10:14343. doi: 10.1038/s41598-020-71001-1 (PMC7459349; doi:10.1038/s41598-020-71001-1)
Supplement: Supplementary file 1 — Supplementary Information 1. [file 41598_2020_71001_MOESM1_ESM.pdf]

Supplementary Information

Title: An Appraisal of Whole-room Indirect Calorimeters and a Metabolic Cart for Measuring Resting and Active Metabolic Rates

Shanshan Chen, Cory Scott, Janina V. Pearce, Jared S. Farrar, Ronald K. Evans, Francesco S. Celi

RMR Study

Figure S1 displays the variability chart of the RMR results. Figure S2 shows the between-instrument biases in a Bland-Altman plot. Figure S3 shows the within-instrument, between-visits biases in a Bland-Altman plot.

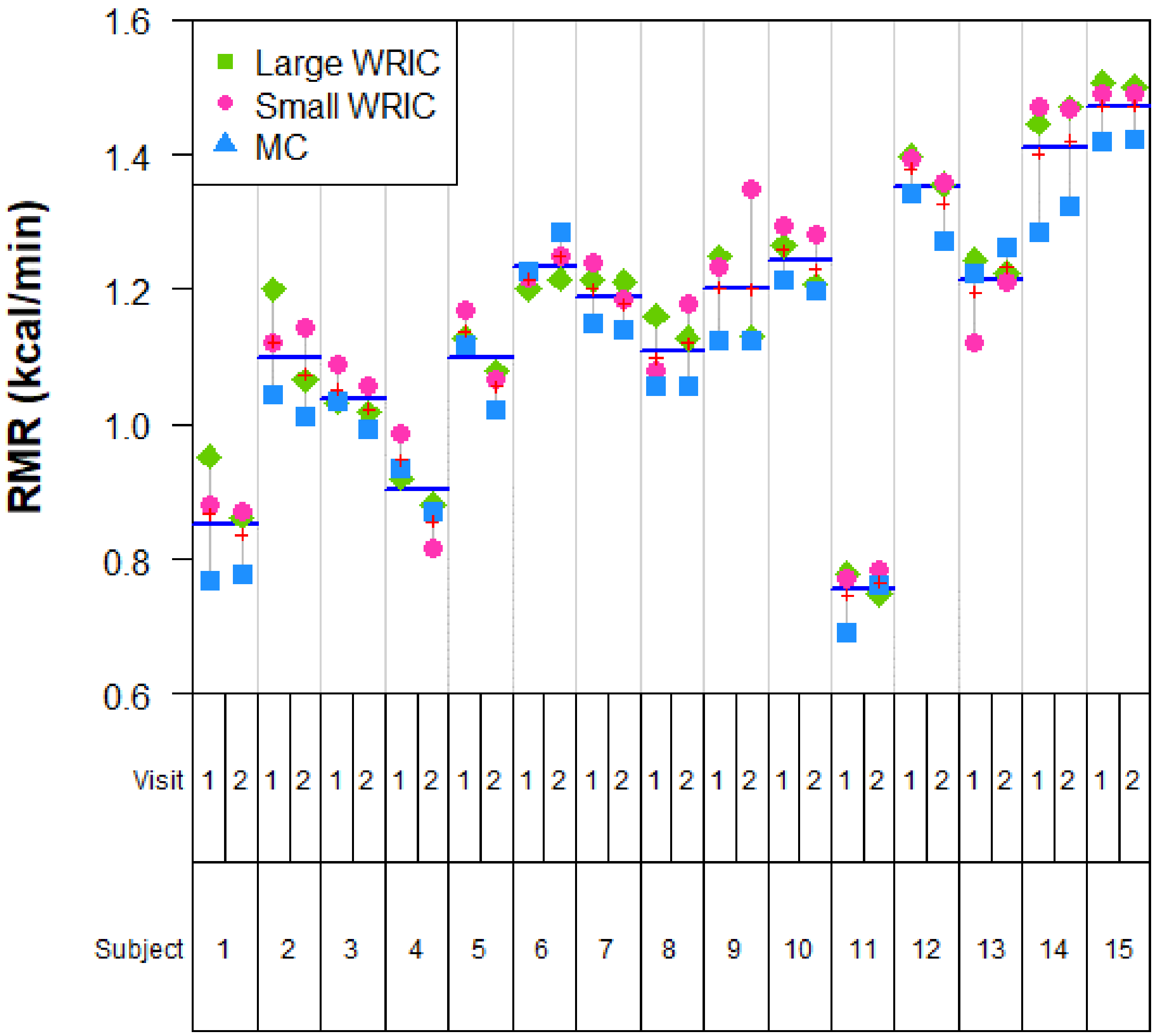

Figure S1 Variability chart of RMR results. Blue lines indicate the average AMR levels across instruments and visits per subject. Red lines indicate the average AMR levels across instruments per subject per visit.

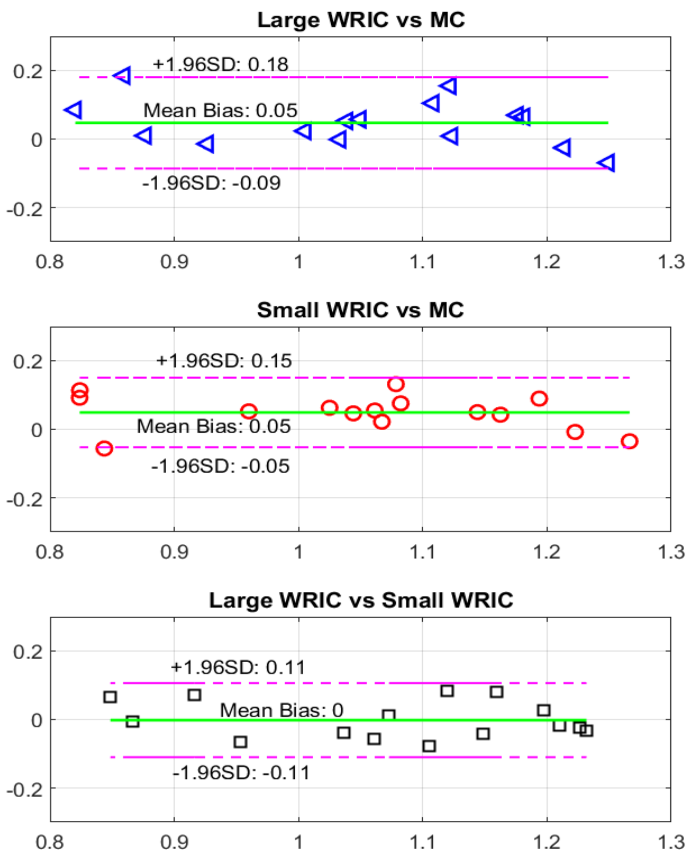

Figure S2 Bland-Altman analysis of the agreement between the three instruments

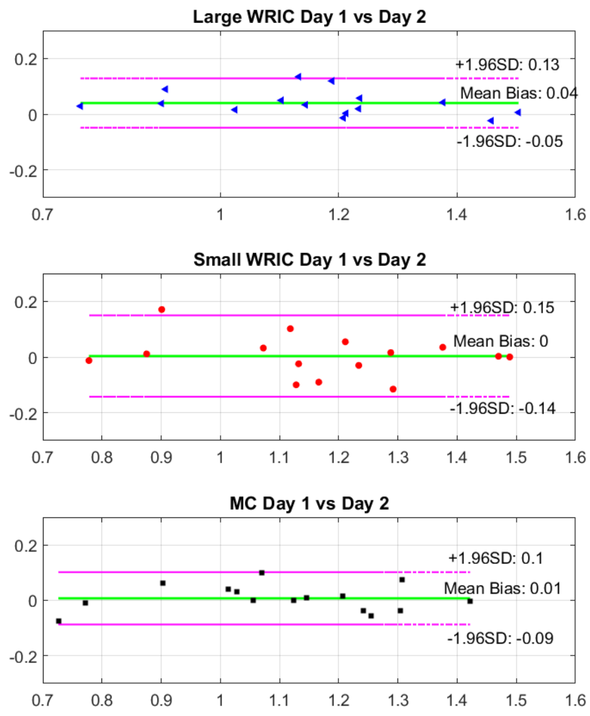

Figure S3 Bland-Altman plot of the RMR measurements on Day 1 and Day 2

AMR Study

Figure S4 displays the variability chart of the AMR results. Figure S5 displays between-instrument biases at each intensity level using Bland-Altman plots in the AMR study.

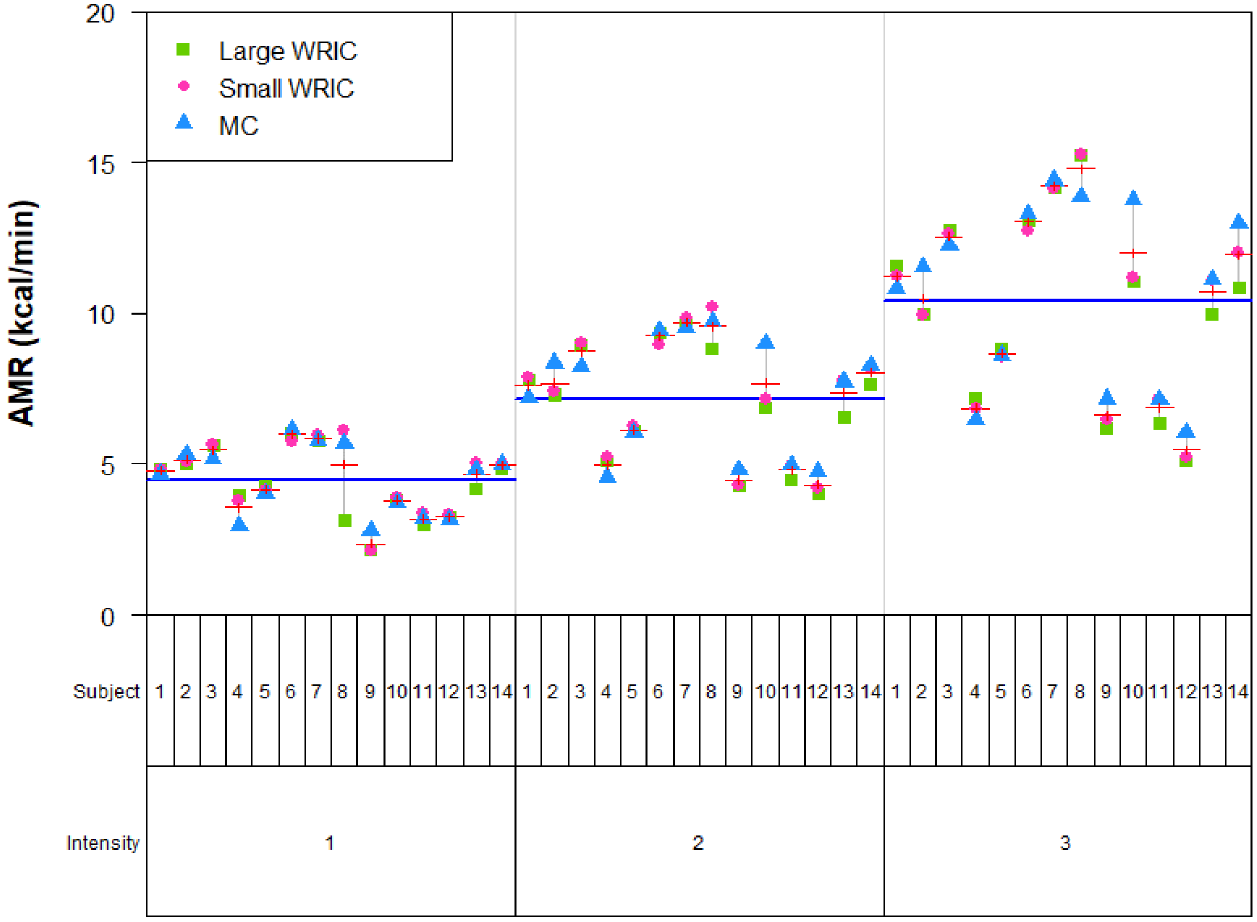

Figure S4 Variability chart of the AMR results. Blue lines indicate the average AMR levels across subjects and instruments at each intensity level. Red lines indicate the average AMR levels across instruments per subject at each intensity level. Except for a few subjects (Subjects 4, 8, 9 at Intensity 1, and Subject 10 at Intensities 2 and 3), the three types of dot representing the three instruments are close together, suggesting that variation by type of instrument is small.

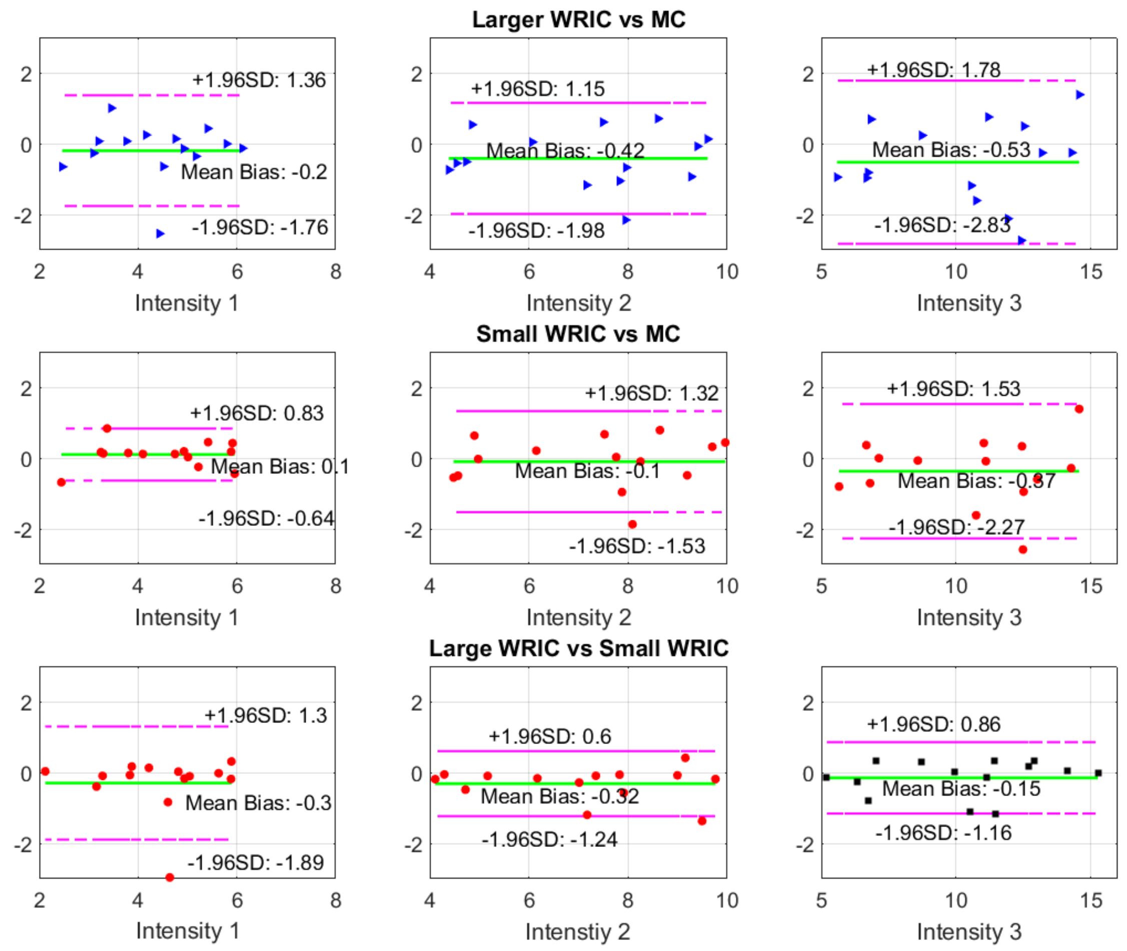

Figure S5 Bland-Altman analysis of the agreement between the three instruments for each exercise intensity

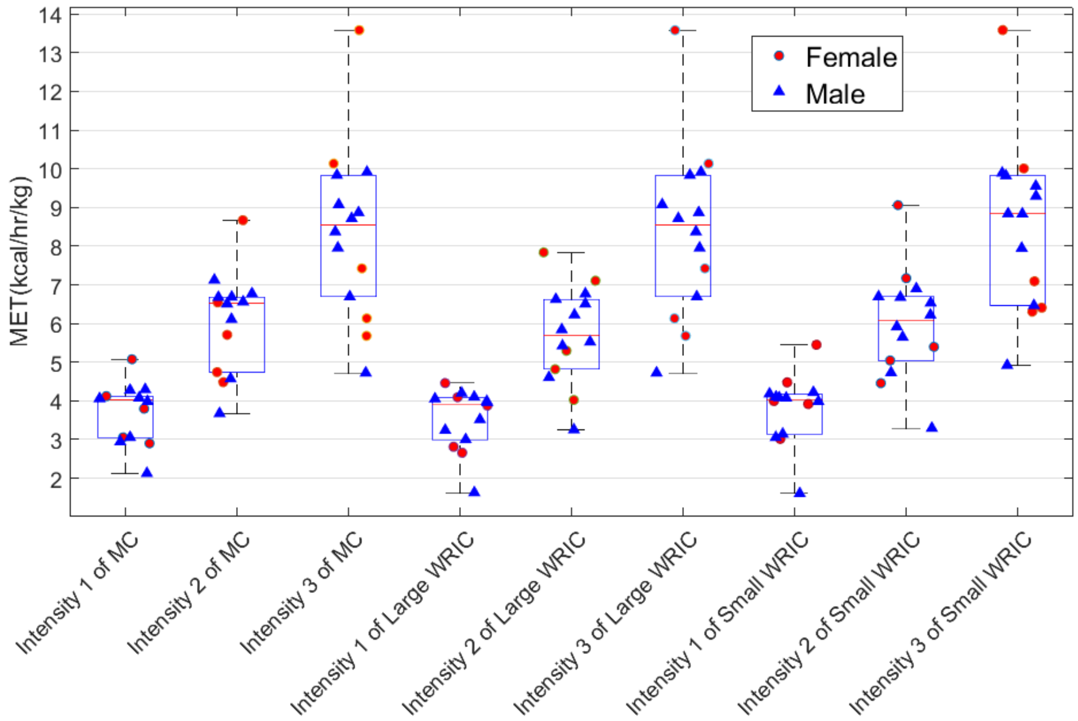

Figure S6 METs at three exercise intensities.

Figure S6 shows the MET range in our AMR protocol. All three instruments showed similar Metabolic Equivalent Task (MET) ranges across three intensity levels, covering moderate (MET $\geq$ 3 but <6 kcal/hr/kg) to vigorous (MET $\geq$ 6 kcal/hr/kg) exercise, with the exception of one subject whose MET value during the lowest exercise intensity was <3 kcal/kg/hr).
